# Supplementary material for: NODULIN HOMEOBOX is required for heterochromatin homeostasis in Arabidopsis
Source: Nat Commun. 2022 Aug 27;13:5058. doi: 10.1038/s41467-022-32709-y (PMC9420119; doi:10.1038/s41467-022-32709-y)
Supplement: Supplementary file 1 — Supplementary Information [file 41467_2022_32709_MOESM1_ESM.pdf]

Supplementary Figures

NODULIN HOMEOBOX is required for heterochromatin homeostasis in *Arabidopsis*

Zsolt Karányi, Ágnes Mosolygó-L, Orsolya Feró, Adrienn Horváth, Beáta Boros-Oláh, Éva Nagy, Szabolcs Hetey, Imre Holb, Henrik Szaker, Márton Miskei, Tibor Csorba, Lóránt Székvölgyi

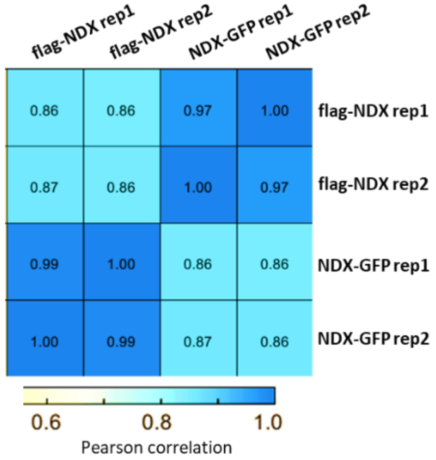

Supplementary Figure 1.

Correlation of flag-NDX and NDX-GFP ChIP-seq data. Numbers show the Pearson correlation coefficient of the datasets. Rep1 and rep2 stand for independent biological replicates.

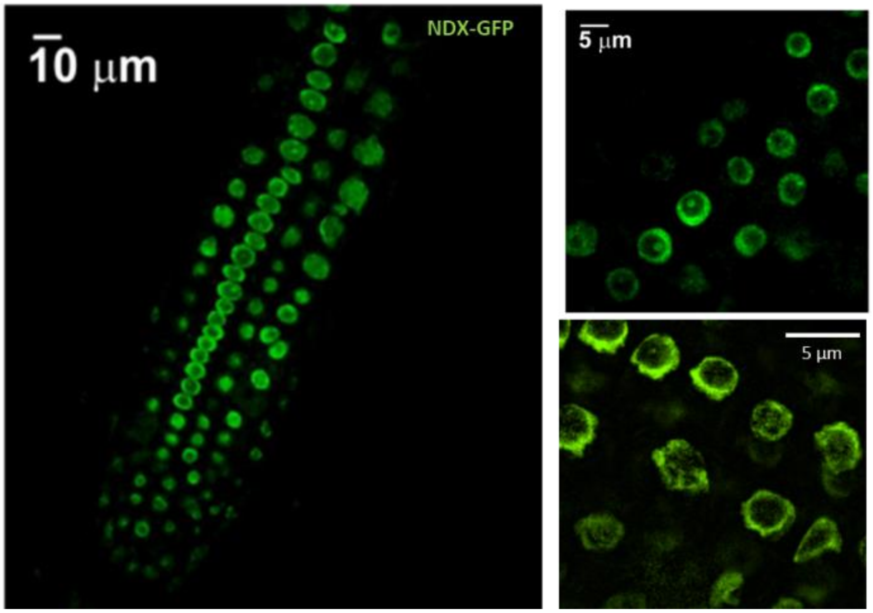

Supplementary Figure 2.

NDX-GFP distribution in *Arabidopsis* root tip nuclei. Root tips from 10-day-old seedlings were imaged at different magnifications using confocal microscopy (representative images are shown). The experiments were repeated 4 times with similar results.

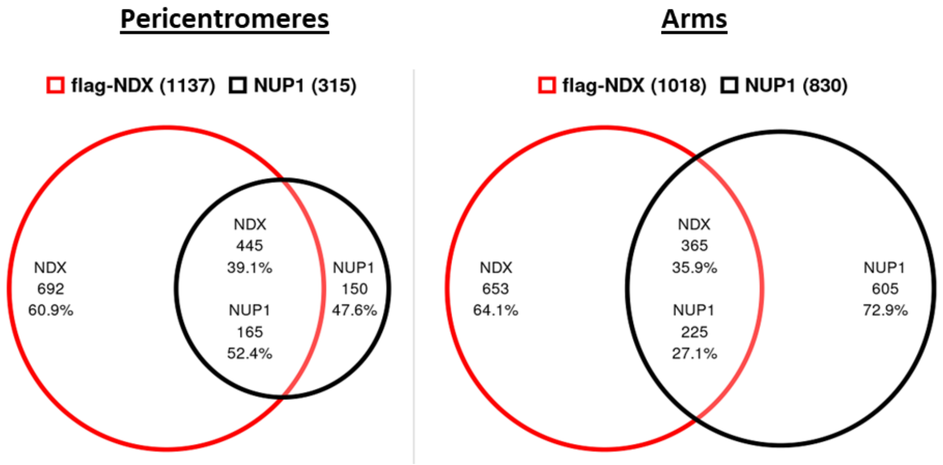

Supplementary Figure 3.

Overlap of NDX and NUCLEOPROIN1 (NUP1) binding sites marking peripheral heterochromatin. In pericentromeric regions, 52.4% of NUP1 peaks colocalize with NDX, which is reduced to 27.1% in chromosome arms (prop.test, *p<0.0001). Flag-NDX peaks were extended from the peak summits to 500 bp (each peak was 500 bp). Pericentromers were defined as CEN positions +/- 2.5 Mb. NUP1 peaks were downloaded from Bi X *et al.* (Genome Res 2017).

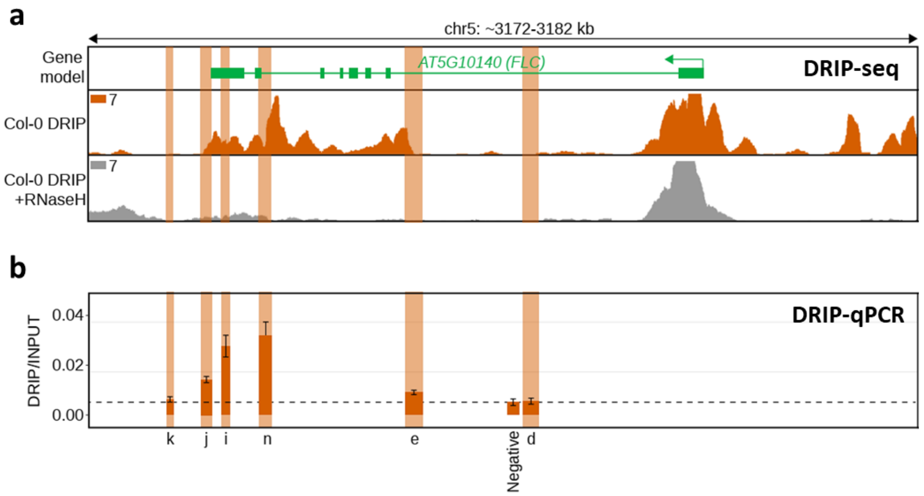

Supplementary Figure 4.

Representative genome browser track showing RNA-DNA hybrid enrichment over the *FLC* (AT5G10140) locus. (a) DRIP-seq and RNase H DRIP-seq. RNase H-treatment represents the background of S9.6 immunoprecipitation. (b) Bar chart showing the DRIP-qPCR validation of peak predictions. Y axis: IP / input. Error bar: SEM. Position of qPCR amplicons (from Sun Q et al. 2013) are highlighted. Sample size n=2 biologically independent experiments.

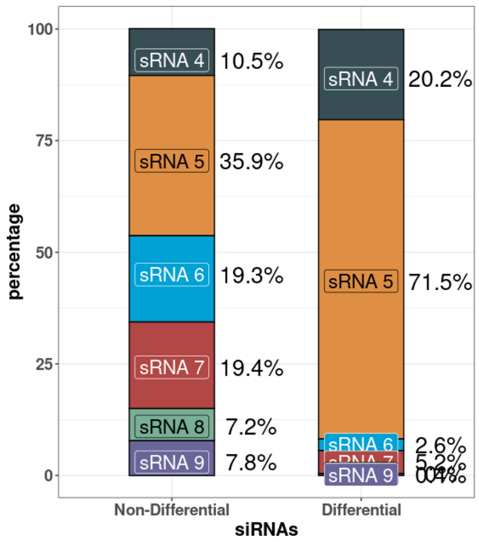

Supplementary Figure 5.

Most differentially expressed sRNAs in the *ndx1-4* mutant belong to sRNA classes 4-9 and thus depend on Pol IV activity. Differential sRNA expression in the ndx1-4 mutant significantly shifts toward sRNA classes 4 and 5 such that their proportion doubles compared to non-differential ones. (We note that sRNA classes 4,5 are typically Pol IV and Pol V dependent, while sRNA classes 6,8,9 depend on Pol IV only (Hardcastle 2018)). These associations demonstrate that Pol IV-derived / dependent siRNAs are specifically altered in the *ndx1-4* mutant.

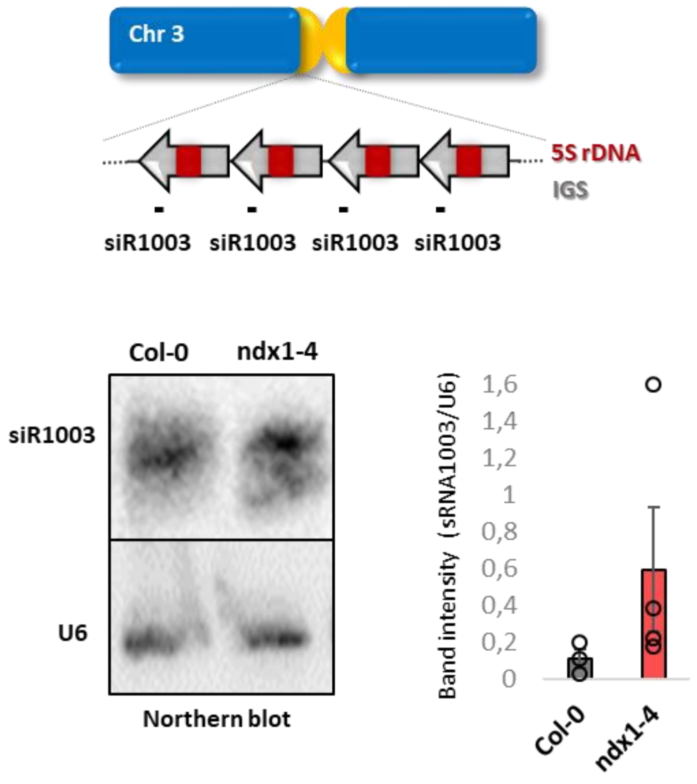

Supplementary Figure 6.

The effect of *ndx1-4* mutation on the sRNA expression profile of 5S rRNA genes located in pericentromeric regions. siR1003 is a small non-coding RNA derived from the intergenic spacers (IGSs) of tandemly repeated 5S rRNA genes whose expression is controlled by non-CG methylation. The northern blot probe used in our experiment represents the silenced 5S rDNA array located at the pericentromere of chr3 (cytologically at the chromocenter), just where NDX preferentially binds. 21-24 nt siR1003 expression is mildly upregulated in the *ndx1-4* mutant (p=0.23, Student's t-test, two sided). siR1003 RNA levels were normalized to U6 ncRNA levels. The blot is a representative image from three biologically independent experiments (sample size n=3). Error bar: SD.

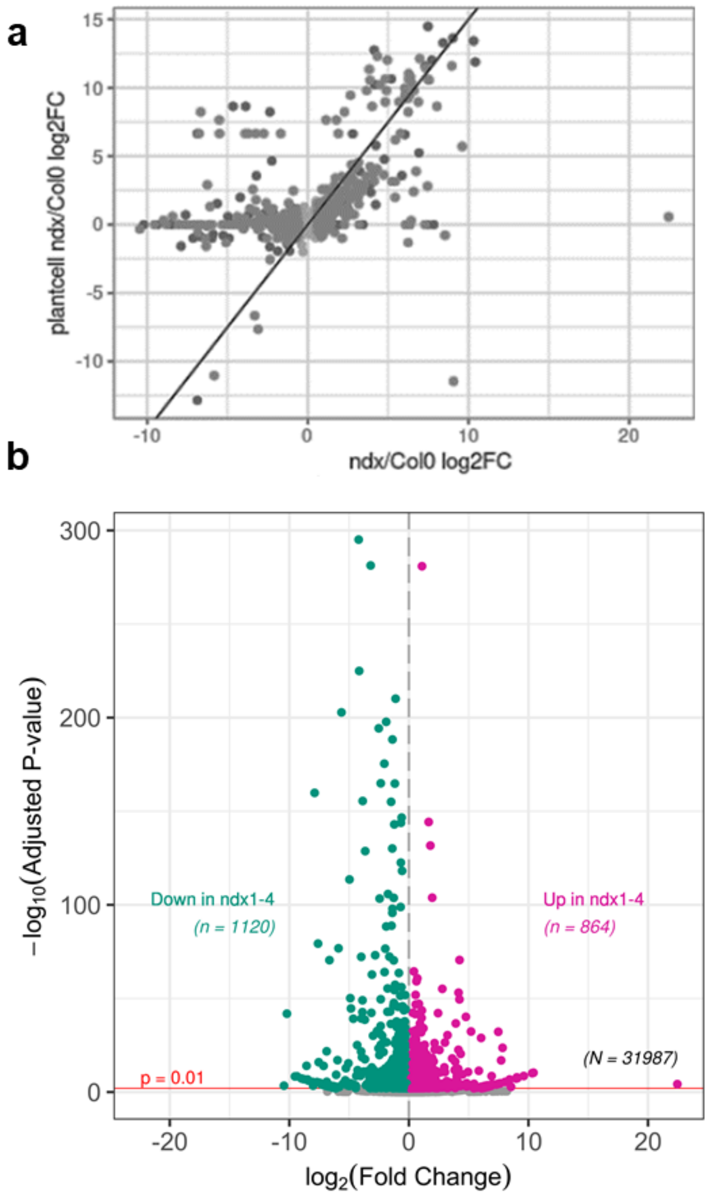

Supplementary Figure 7.

Differential mRNA expression in the *ndx1-4* mutant. (a) Correlation of gene expression changes between two ndx1-4 mRNA-seq datasets. Y axis: differentially expressed genes (DEGs) identified in the Zhu *et al.* dataset (Plant Cell 2020); *ndx1-4*/Col-0 $\log_2$ fold change. X axis: DEGs identified in the current dataset (Karanyi *et al.*); *ndx1-4*/Col-0 $\log_2$ fold change. The two datasets are significantly correlated (Pearson R = 0.81). (b) Volcano plot representation of significantly down- and up-regulated mRNAs identified by RNA-seq. P-value ≤ 0.01, Wald test; p-value adjustment: Benjamini & Hochberg (FDR) method. Sample size n=2 biologically independent replicates.

AgriGO - Downregulated Genes

GO_acc	Term	queryitem	bgitem	pvalue	FDR
GO:0006950	response to stress	296	3506	9.8E-37	1.2E-33
GO:0048856	anatomical structure development	170	3146	0.000012	0.00045
GO:0032553	ribonucleotide binding	139	2664	0.00036	0.0083
GO:0009791	post-embryonic development	93	1576	0.000085	0.0025
GO:0006629	lipid metabolic process	88	994	8E-12	9.3E-10
GO:0033993	response to lipid	72	772	8.3E-11	8.2E-09
GO:0048316	seed development	39	661	0.0093	0.14
GO:0008289	lipid binding	34	317	4.9E-07	0.00004
GO:0048364	root development	31	482	0.0065	0.1
GO:0010876	lipid localization	29	199	9.9E-09	7.2E-07

AgriGO - Upregulated genes

GO_acc	Term	queryitem	bgitem	pvalue	FDR
GO:1990904	ribonucleoprotein complex	58	852	2E-08	7.6E-07
GO:0099402	plant organ development	43	961	0.0071	0.25
GO:0009409	response to cold	42	384	5.5E-12	2.6E-09
GO:0003735	structural constituent of ribosome	37	391	4E-09	1.5E-06
GO:0048367	shoot system development	36	896	0.049	0.91
GO:0044391	ribosomal subunit	32	322	1.5E-08	6.7E-07
GO:0003729	mRNA binding	24	337	0.00015	0.013
GO:0009908	flower development	24	520	0.028	0.65
GO:0048437	floral organ development	18	230	0.00035	0.025
GO:0001012	RNAPII regulatory region DNA binding	9	90	0.0023	0.1

Supplementary Figure 8.

Gene Ontology (GO) term annotation of differentially expressed mRNA coding genes using AgriGO. Statistics: Fisher's exact test. P-values are computed and adjusted by multiple testing corrections using the Benjamini & Hochberg (FDR) method.

Differentially expressed TEs detected by mRNA-seq

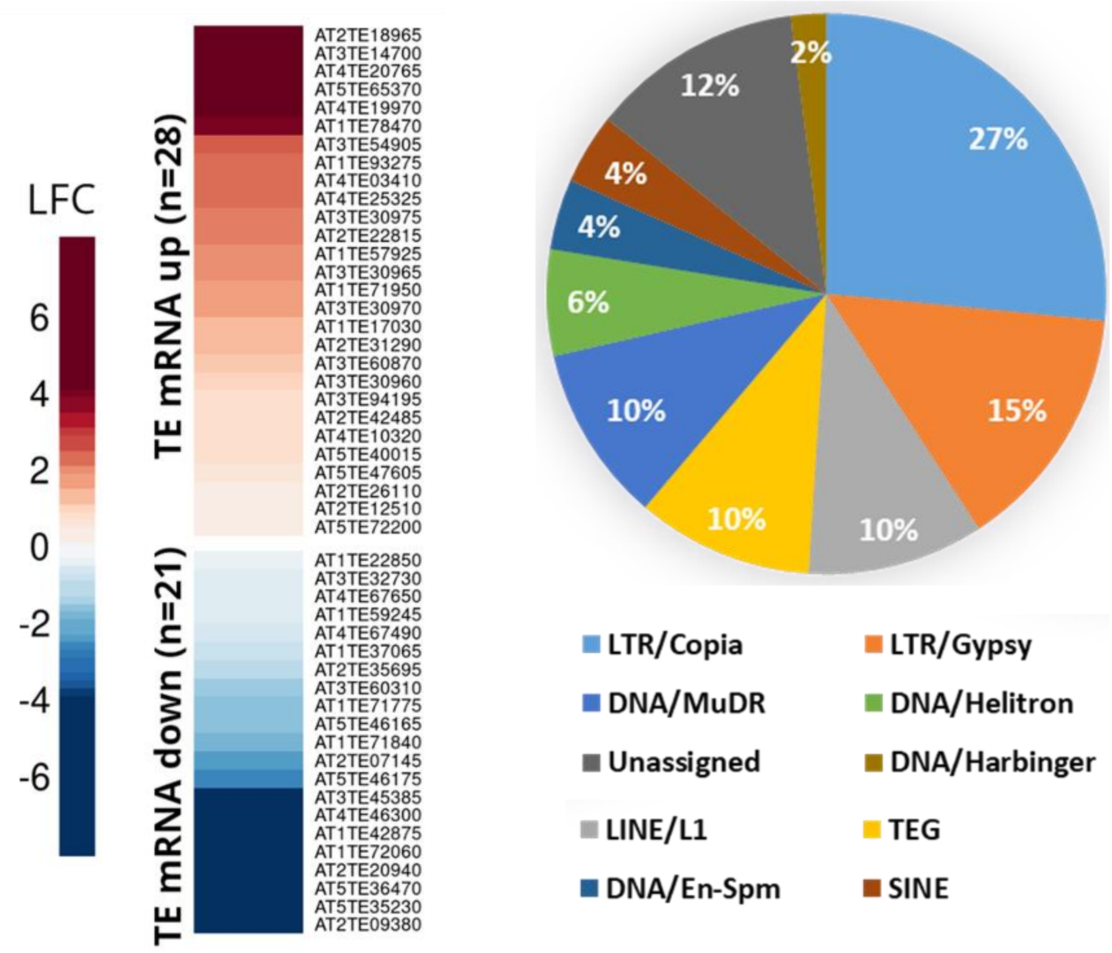

Supplementary Figure 9.

Loss of NDX function weakly affects transposon activity detected by mRNA-seq. (left) Heatmap of differentially expressed mRNAs in Col-0 and ndx1-4 seedlings detected by mRNA-seq. Statistical significance: $p<0.05$; permutation test; p-value adjustment: Benjamini & Hochberg (FDR) method. Colour heat is proportional to DeSeq2 log fold-change (LFC). (right) Distribution of differentially expressed TEs in transposon superfamilies. Retrotransposons (LTR/Copia, LTR/Gypsy, LINE1) represent more than 50% of the cases. We note that 20 TEs overlapped with protein-encoding ORFs and were not excluded from the analysis.

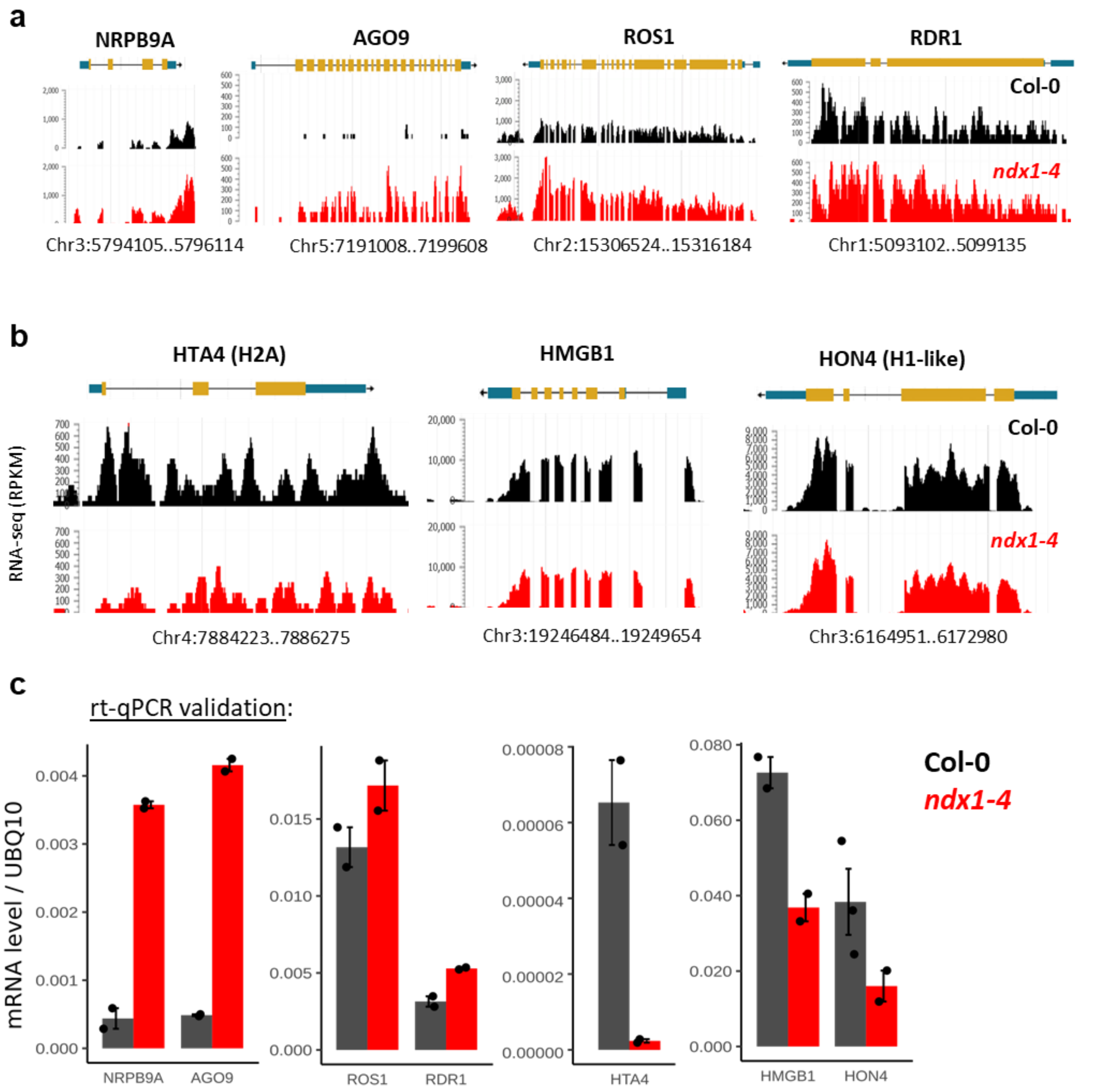

Supplementary Figure 10.

Chromatin regulators showing differential mRNA expression in *ndx1-4* mutants. (a-b): Genome browser snapshots of upregulated (a) and downregulated (b) chromatin regulator genes. (c) RT-qPCR validation of gene expression changes shown in panels a-b. mRNA levels were normalized with UBQ10 expression levels. Error bar: SEM. Sample size n=2 biologically independent experiments.

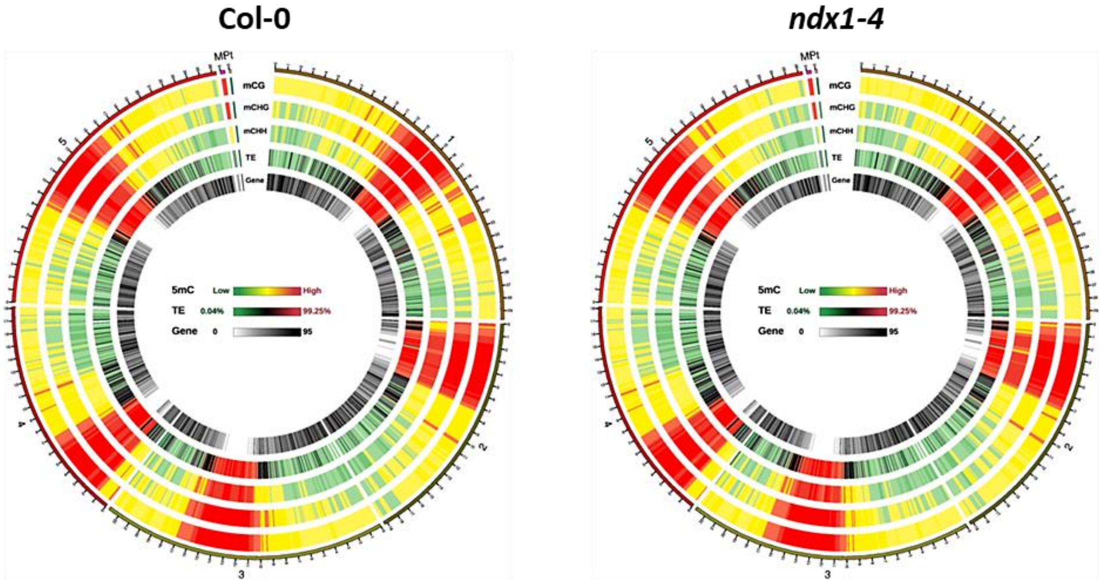

Supplementary Figure 11.

Circos plot showing genome-wide CG/CHG/CHH methylation densities in Col-0 and *ndx1-4* plants. Heatmaps from outside to inside: 1. CG methylation density 2. CHG methylation density 3. CHH methylation density 4. T (transposon) density 5. Gene density.

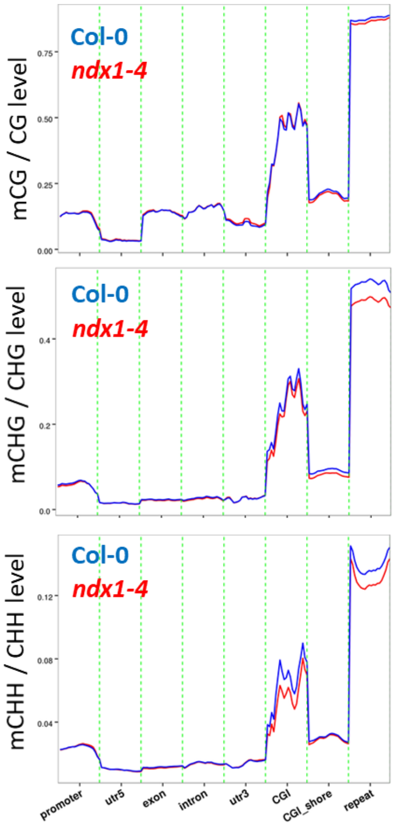

Supplementary Figure 12.

Methylation level distribution at functional genetic elements in each context (CG, CHG, CHH). Each functional region is divided into 20 bins and methylation levels are calculated in each bin.

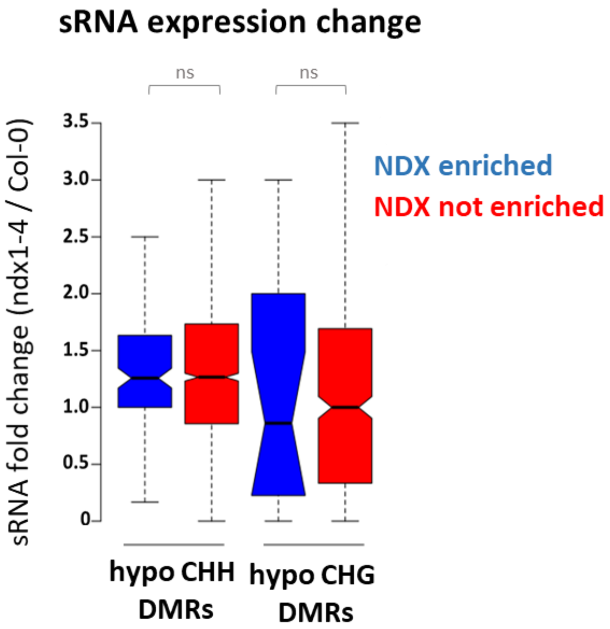

Supplementary Figure 13.

sRNA expression changes at hypo CHH and CHG DMRs identified in the *ndx1-4* mutant. Hypo CHH/CHG DMRs were classified as NDX enriched (blue) and non-enriched (red) based on their overlap with flag-NDX ChIP binding sites, detected in Col-0 plants. Y-axis represents sRNA-seq fold change values in *ndx1-4* relative to Col-0. There is no statistically significant difference between NDX-enriched and non-enriched DMRs (Wilcoxon rank sum test, two sided). Bounds of boxes describe the interquartile range with the median; whiskers indicate minimum and maximum values; outliers are not shown.

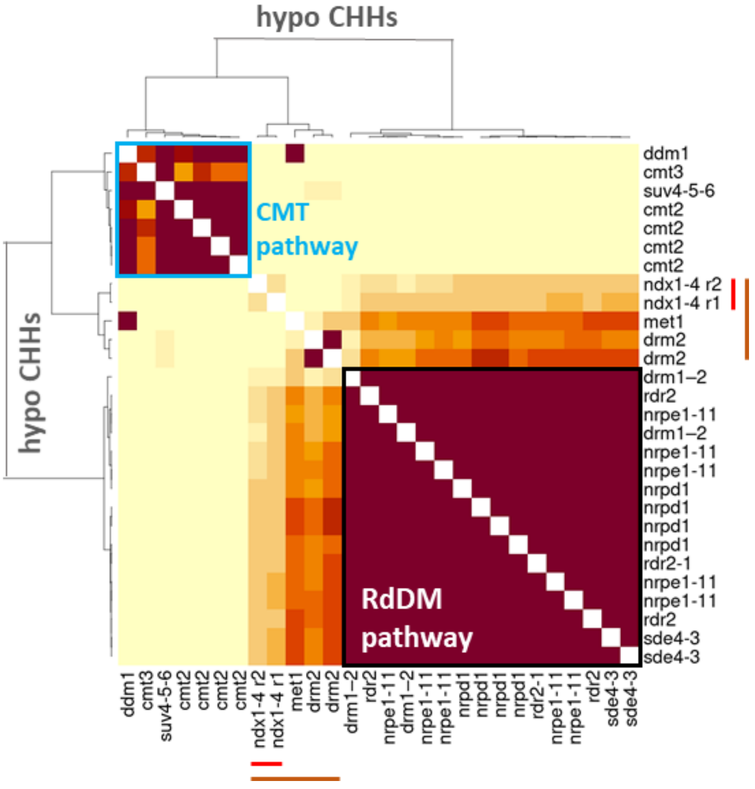

Supplementary Figure 14.

Clustering of hypo CHH DMRs identified by BS-seq in different DNA methylation mutants. Using the hcDMR pipeline (Zhang *et al.* 2018 PNAS), we compared BS-seq libraries with more than 50 high-quality Col-0 controls and defined high-confidence differentially methylated regions (hcDMRs) in the CHH context. HcDMRs were clustered with the S-MOD method (statistical measurement of overlapping of DMRs), allowing the identification of hierarchical relationships between *ndx1-4* and DNA methylation mutants The *ndx1-4* mutant (highlighted by red lines) clusters with the *drm2*, and *met1* mutants (brown lines).

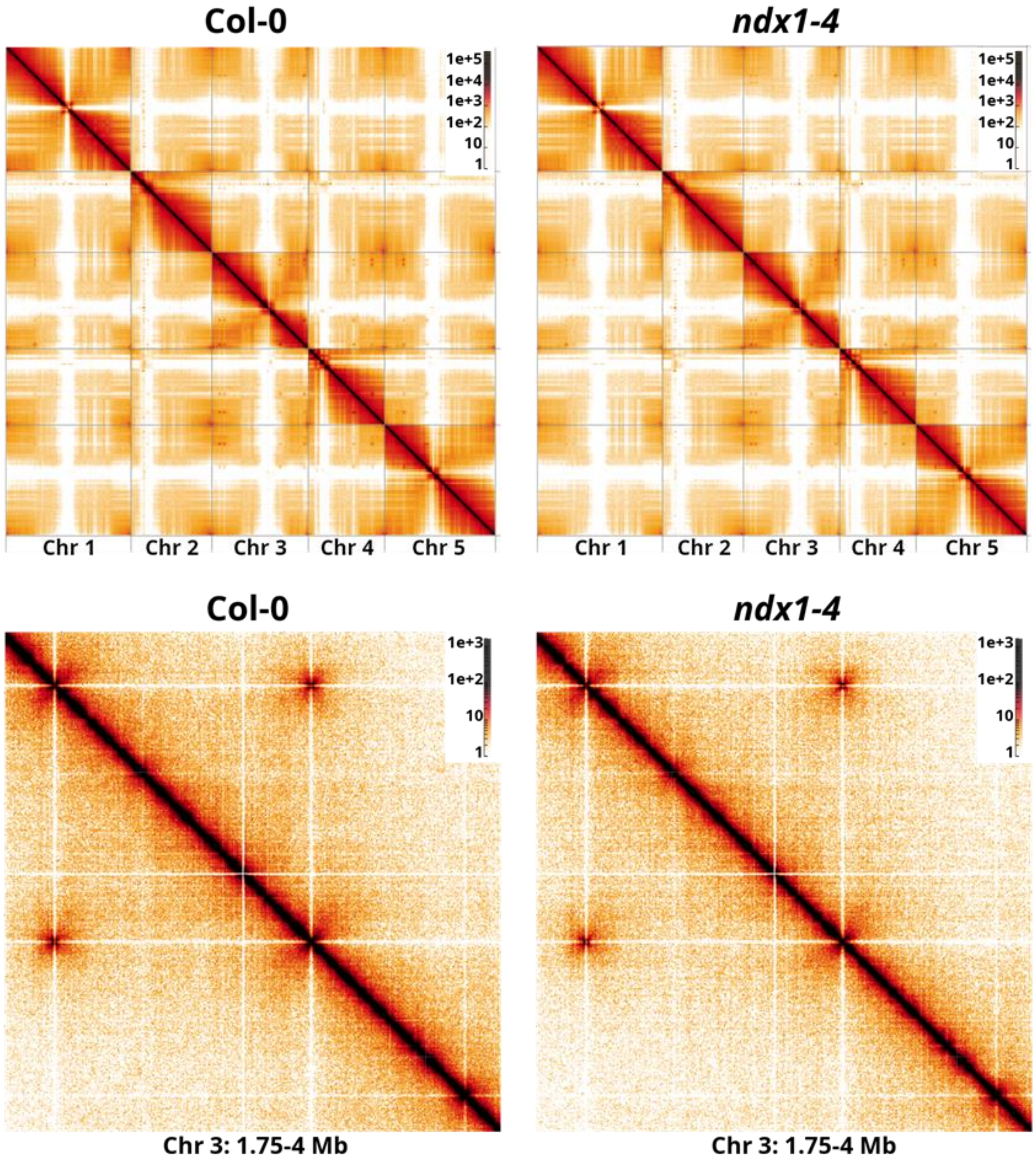

Supplementary Figure 15.

Hi-C interaction maps in Col-0 and *ndx1-4* seedlings. Interaction matrices are shown for the full genome or selected chromosomal regions. Resolution: 25 kb.

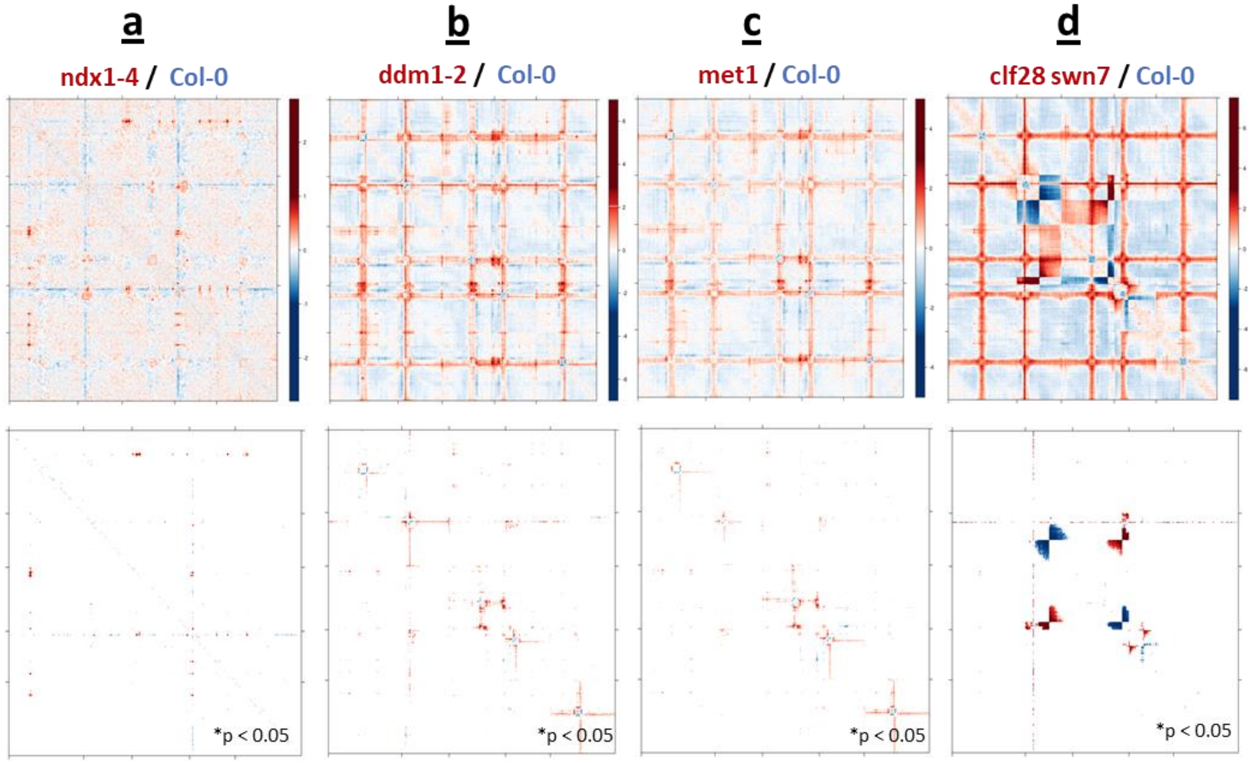

Supplementary Figure 16.

Loss of NDX causes similar genome structural changes as DNA methylation mutants. Upper panels: Differential Hi-C interaction matrix of *ndx1-4, ddm1-2, met1-3, and clf28 swn7* mutants versus Col-0. Fold change, $\log_2$ mutant (red) / Col-0 (blue). Red and blue colours show enrichment or depletion of Hi-C interactions in the mutants, respectively. White colour indicates no change. Resolution: 25 kb. Lower panels: Regions showing statistically significant differences in Hi-C interactions identified by the Slitherine algorithm (statistical significance: $p<0.05$; permutation test; p-value adjustment: Benjamini & Hochberg (FDR) method.)

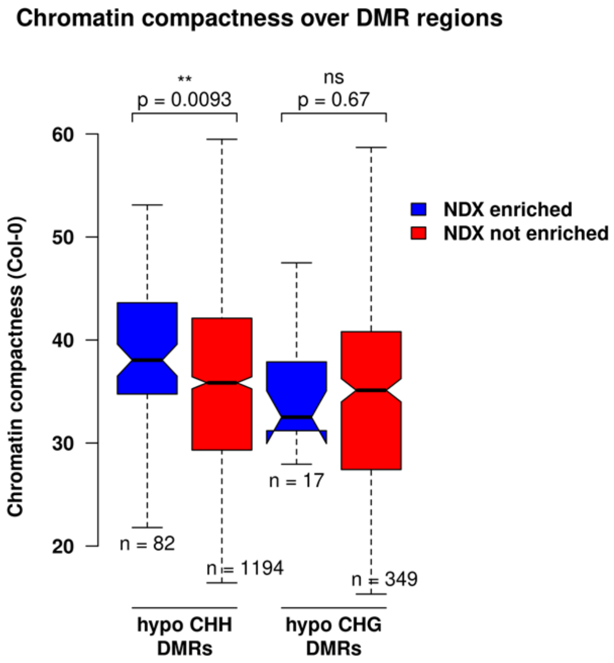

Supplementary Figure 17.

Chromatin compactness changes of hypo CHH and CHG DMRs identified in *ndx1-4* mutant. Hypo CHH/CHG DMRs were classified as NDX enriched (blue) and non-enriched (red) based on their overlap with flag-NDX ChIP binding sites, detected in Col-0 plants. Sample size (n) = number of DMRs examined over 2 independent experiments. Bounds of boxes describe the interquartile range with the median; whiskers indicate minimum and maximum values; outliers are not shown. Y axis shows compactness scores calculated from Hi-C data. At hypo CHH DMRs there is statistically significant difference between NDX-enriched and non-enriched sites (**p=0.0093, Wilcoxon rank sum test, two sided; p-value adjustment: Holm method).

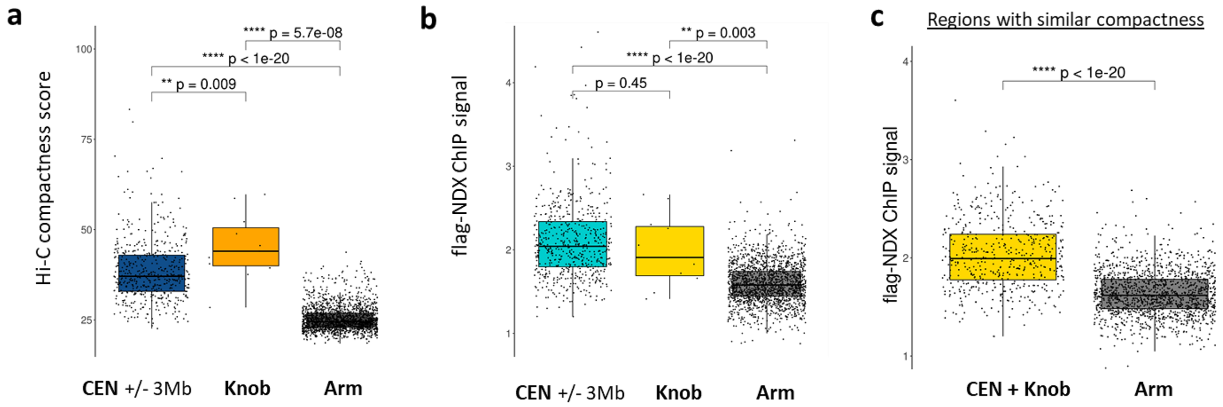

Supplementary Figure 18.

The relationship between chromatin compactness and NDX binding. (a) Distribution of compactness scores calculated from Hi-C data. Pericentromeric, Knob (Chr4:1,560,561-2,082,885; PMID: 32312999), and chromosome arm regions are compared in 50 kb bins. (b) Distribution of NDX ChIP signal (average values in 50 kb bins) over pericentromeric, Knob and chromosome arm regions. (c) Distribution of NDX ChIP signal over genomic regions with similar Hi-C compactness, randomly sampled from pericentromeric, Knob and arm regions. CEN+Knob regions were merged since the number of relevant bins were very low in the Knob region to assign statistical significance (Wilcoxon rank sum test (two-sided); p-value adjustment: Holm method). Bounds of boxes describe the interquartile range with the median; whiskers indicate minimum and maximum values; outliers are not shown.

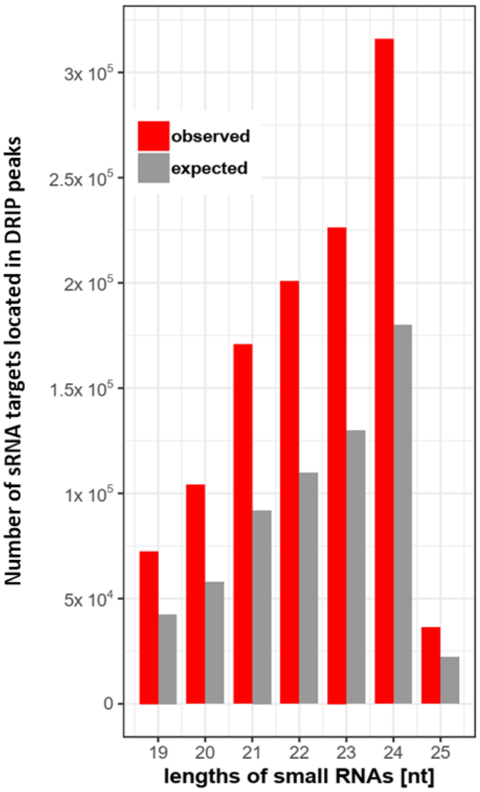

Supplementary Figure 19.

The number of sRNA-targets over R-loops is significantly higher than expected by chance. Distribution of sRNA-targets 19-25 nt sRNA-targets over R-loops (red) and random sites (grey) that show differential expression in *ndx1-4*. R-loops represent ectopic targets for siRNAs.
