# Supplementary material for: NODULIN HOMEOBOX is required for heterochromatin homeostasis in Arabidopsis
Source: Nat Commun. 2022 Aug 27;13:5058. doi: 10.1038/s41467-022-32709-y (PMC9420119; doi:10.1038/s41467-022-32709-y)
Supplement: Supplementary file 3 — Description of Additional Supplementary Files [file 41467_2022_32709_MOESM3_ESM.pdf]

Description of Additional Supplementary Files

File Name: Supplementary Data 1
Description: Description of NGS samples

File Name: Supplementary Data 2
Description: NDX binding sites (ChIP peaks) identified in A. thaliana flag-NDX seedling

File Name: Supplementary Data 3
Description: NDX binding sites (ChIP peaks) identified in A. thaliana NDX-GFP seedling

File Name: Supplementary Data 4
Description: RNA-DNA hybrids (DRIP peaks) identified in A. thaliana wild-type (Col-0) seedling

File Name: Supplementary Data 5
Description: RNA-DNA hybrids (DRIP peaks) identified in A. thaliana ndx1-4 mutant seedling
H-4032 Debrecen, Egyetem tér 1., H-4002 Debrecen, P.O.B. 400. tel.: 36-52-416-432, fax: 36-52-314-989

File Name: Supplementary Data 6
Description: Differentially expressed sRNA loci (A. thaliana, ndx1-4 mutant vs. Col-0 wild type seedling)

File Name: Supplementary Data 7
Description: Differentially expressed miRNAs (A. thaliana, ndx1-4 mutant vs. Col 0 wild type seedling)

File Name: Supplementary Data 8
Description: Differentially expressed genes (A. thaliana, ndx1-4 mutant vs. Col-0 wild type seedling)

File Name: Supplementary Data 9
Description: Differentially expressed transposable elements (A. thaliana, ndx1-4 mutant vs. Col-0 wild type seedling)

File Name: Supplementary Data 10
Description: Bisulfite sequencing (BS-seq) raw data summary

File Name: Supplementary Data 11
Description: Differentially methylated regions (DMR)

File Name: Supplementary Data 12
Description: Hi-C alignment statistics

File Name: Supplementary Data 13
Description: Differential Hi-C interactions

File Name: Supplementary Data 14
Description: Oligos used for qPCR validation and northern blot hybridization

File Name: Supplementary Data 15
Description: External datasets

File Name: Supplementary Data 16
Description: Contents of Supplementary Data 1-15 accompanying the paper
