# Supplementary material for: NODULIN HOMEOBOX is required for heterochromatin homeostasis in Arabidopsis
Source: Nat Commun. 2022 Aug 27;13:5058. doi: 10.1038/s41467-022-32709-y (PMC9420119; doi:10.1038/s41467-022-32709-y)
Supplement: Supplementary file 20 — Reporting Summary [file 41467_2022_32709_MOESM20_ESM.pdf]

nature portfolio

Corresponding author(s): Lorant Szekvolgyi

Last updated by author(s): Aug 9, 2022

Reporting Summary

Nature Portfolio wishes to improve the reproducibility of the work that we publish. This form provides structure for consistency and transparency in reporting. For further information on Nature Portfolio policies, see our Editorial Policies and the Editorial Policy Checklist.

Statistics

For all statistical analyses, confirm that the following items are present in the figure legend, table legend, main text, or Methods section.

n/a	Confirmed	
☐	☒	The exact sample size (*n*) for each experimental group/condition, given as a discrete number and unit of measurement
☐	☒	A statement on whether measurements were taken from distinct samples or whether the same sample was measured repeatedly
☐	☒	The statistical test(s) used AND whether they are one- or two-sided<br>*Only common tests should be described solely by name; describe more complex techniques in the Methods section.*
☒	☐	A description of all covariates tested
☐	☒	A description of any assumptions or corrections, such as tests of normality and adjustment for multiple comparisons
☐	☒	A full description of the statistical parameters including central tendency (e.g. means) or other basic estimates (e.g. regression coefficient) AND variation (e.g. standard deviation) or associated estimates of uncertainty (e.g. confidence intervals)
☐	☒	For null hypothesis testing, the test statistic (e.g. *F*, *t*, *r*) with confidence intervals, effect sizes, degrees of freedom and *P* value noted<br>*Give P values as exact values whenever suitable.*
☒	☐	For Bayesian analysis, information on the choice of priors and Markov chain Monte Carlo settings
☒	☐	For hierarchical and complex designs, identification of the appropriate level for tests and full reporting of outcomes
☒	☐	Estimates of effect sizes (e.g. Cohen's *d*, Pearson's *r*), indicating how they were calculated

Our web collection on statistics for biologists contains articles on many of the points above.

Software and code

Policy information about availability of computer code

Data collection	QuantStudio 12K Flex Real-Time PCR System (Thermo Fisher Scientific) to collect qPCR data<br>Olympus FluoView 1000 confocal microscope for FCS, FRAP and CLSM measurements<br>Molecular Imager PharosFX Systems for 32P signal detection in northern blot experiments<br>Illumina NextSeq500 for NGS sequencing<br>Illumina HiSeq 2500 for NGS sequencing<br>Agilent bioanalyzer for capillary electrophoresis of NGS libraries, DNA preps and RNA preps (quality control)
Data analysis	QuickFit 3.0 for autocorrelation analysis (FCS experiments)<br>bowtie2 2.3.5.1 for ChIP-seq, DRIP-seq alignments<br>samtools 1.7 for making .bam files and indexing in ChIP-seq, DRIP-seq experiments<br>picard 2.13.2 for filtering low mapping quality and PCR-duplicated reads in NGS experiments<br>deeptools 3.3.1 to create RPKM (Reads Per Kilobase per Million mapped reads) normalized coverage files (.bedgraph and .bigwig)<br>MACS v2.2.7.1 to identify ChIP and DRIP peaks<br>bedtools v2.27.1 for genomic annotation of DRIP and ChIP peaks<br>R-project v4.1. for statistical calculations and image generations<br>mfold 3.6 for ssCount calculations<br>sRNAnalyzer for sRNA-seq lignment and analysis<br>Cutadapt 3.4 to trim Illumina adapters in NGS experiments<br>psRNATarget for sRNA target site prediction<br>Salmon 1.7.0 to map mRNA-seq reads and get transcript quantities for genes and transposable elements<br>DESeq2 1.24.0 to identify differentially expressed genes and TEs<br>HISAT2 2.2.1 for RNA-seq read alignment to the TAIR10 reference genome<br>agriGO for gene ontology enrichment analysis of differentially expressed protein coding genes<br>ImageJ 1.52r for microscopic image analysis

Bismark v0.22.3 to align the bisulfite-treated reads to the TAIR10 reference genome and for the analysis of BS-seq
hcDMR caller to identify high confidence differentially methylated regions in a larg collection of epigenetic mutants, inculding ndx1-4
Serpentine to smooth local noise in Hi-C interaction maps and identifies statistically significant differences between Hi-C contact matrices
Slitherine to smooth local noise in Hi-C interaction maps and identifies statistically significant differences between Hi-C contact matrices
JBrowse 1.6.11 for genome browser representation of NGS data
Juicer tools 1.22.01 for Hi-C analysis
HiGlass v1.11.7 for generating publication ready images of Hi-C interaction matrices

For manuscripts utilizing custom algorithms or software that are central to the research but not yet described in published literature, software must be made available to editors and reviewers. We strongly encourage code deposition in a community repository (e.g. GitHub). See the Nature Portfolio guidelines for submitting code & software for further information.

Data

Policy information about availability of data

All manuscripts must include a data availability statement. This statement should provide the following information, where applicable:

- Accession codes, unique identifiers, or web links for publicly available datasets
- A description of any restrictions on data availability
- For clinical datasets or third party data, please ensure that the statement adheres to our policy

Datasets generated for this study can be accessed in Supplementary Data 1-16 and via JBrowse (https://geneart.med.unideb.hu/pub/2021-ndx). Raw data are available at GEO (GSE201841). External datasets: TAIR10 gene annotation files (gene list, splice junctions) were obtained from The Arabidopsis Information Resource. Promoter and downstream regions were defined as the arbitrary extension of transcription start sites (TSS) and termination sites (TTS) by 2000 base pairs. All other datasets used in this study are summarized in Supplementary Data 15. Source data are provided with this paper.

Field-specific reporting

Please select the one below that is the best fit for your research. If you are not sure, read the appropriate sections before making your selection.

☒ Life sciences ☐ Behavioural & social sciences ☐ Ecological, evolutionary & environmental sciences

For a reference copy of the document with all sections, see nature.com/documents/nr-reporting-summary-flat.pdf

Life sciences study design

All studies must disclose on these points even when the disclosure is negative.

Sample size	Samples sizes were not predetermined by statistical testing. Sample sizes used to derive statistics were defined and described for each figure.
Data exclusions	During the DRIP-seq and ChIP-seq peak calling we excluded peaks from genomic regions in which the input signal was zero. These peaks were consistently regarded as false positives as were omitted from the final peak list.
Replication	Each experiments have been repeated at least two times (biologically independent replicates). The experimental findings were reproducible.
Randomization	For the functional annotation of DRIP-seq and ChIP-seq peaks over genomic features, observed / expected overlap ratios were determined. The expected (theoretical) distribution was calculated from computer randomized peak sets generating a null distribution to assign statistical significance.
Blinding	Blinding was not applied during the execution of the experiments.

Reporting for specific materials, systems and methods

We require information from authors about some types of materials, experimental systems and methods used in many studies. Here, indicate whether each material, system or method listed is relevant to your study. If you are not sure if a list item applies to your research, read the appropriate section before selecting a response.

Materials & experimental systems

n/a	Involved in the study	
☐	☒	Antibodies
☐	☒	Eukaryotic cell lines
☒	☐	Palaeontology and archaeology
☐	☒	Animals and other organisms
☒	☐	Human research participants
☒	☐	Clinical data
☒	☐	Dual use research of concern

Methods

n/a	Involved in the study	
☐	☒	ChIP-seq
☒	☐	Flow cytometry
☒	☐	MRI-based neuroimaging

Antibodies

Antibodies used	S9.6 monoclonal antibody, prepared in house from the S96 hybridoma cell line, was applied for DRIP. Anti-flag antibody - #2044 New England Biolabs; anti-GFP antibody - #ab290 Abcam, were used for ChIP. Secondary antibody (goat anti-mouse-HRP), Thermo Fisher, was applied for slot blot.
Validation	All antibodies were ChIP grade according to the Manufacturer description. The specificity of S9.6 IP was assessed by RNaseH digestion of gDNA samples before the IP step. The DRIP signal was reduced significantly in all experiments. The flag- and GFP ChIP experiments were always supplemented with "no tag" control samples, which consistently showed a reduced and aspecific signal.

Eukaryotic cell lines

Policy information about cell lines

Cell line source(s)	S9.6 (Hb-8730) mouse hybridoma cell line (Atcc)
Authentication	None of the cell lines used were authenticated.
Mycoplasma contamination	All cell lines tested negative for mycoplasma contamination.
Commonly misidentified lines (See ICLAC register)	-

Animals and other organisms

Policy information about studies involving animals; ARRIVE guidelines recommended for reporting animal research

Laboratory animals	The study did not involve lab animals.
Wild animals	The study did not involve wild animals.
Field-collected samples	The study did not involve samples collected from the field.
Ethics oversight	No ethical approval was necessary for the experiments.

Note that full information on the approval of the study protocol must also be provided in the manuscript.

ChIP-seq

Data deposition

☒ Confirm that both raw and final processed data have been deposited in a public database such as GEO.

☒ Confirm that you have deposited or provided access to graph files (e.g. BED files) for the called peaks.

Data access links
May remain private before publication.

http://geneart.med.unideb.hu/pub/2021-ndx
https://www.ncbi.nlm.nih.gov/geo/query/acc.cgi?acc=GSE201841

Files in database submission

```
#ChIP-seq
"ChIP\ChIP_Meta_2021.xlsx"
"ChIP\at_chip_ndx-gfp_r1.bw"
"ChIP\at_chip_ndx-gfp_r2.bw"
"ChIP\at_chip_flag-ndx_r2.bw"
"ChIP\at_chip_ndx-gfp.bed"
"ChIP\at_chip_flag-ndx_r1.bw"
"ChIP\at_chip_flag-ndx.bed"
"ChIP\at_chip_flag-ndx_ip_r2_2.fastq.gz"
"ChIP\at_chip_flag-ndx_ip_r2_1.fastq.gz"
"ChIP\at_chip_flag-ndx_ip_r1_2.fastq.gz"
"ChIP\at_chip_flag-ndx_ip_r1_1.fastq.gz"
"ChIP\at_chip_flag-ndx_inp_2.fastq.gz"
"ChIP\at_chip_flag-ndx_inp_1.fastq.gz"
"ChIP\at_chip_ndx-gfp_ip_r2_2.fastq.gz"
"ChIP\at_chip_ndx-gfp_ip_r2_1.fastq.gz"
"ChIP\at_chip_ndx-gfp_ip_r1_2.fastq.gz"
"ChIP\at_chip_ndx-gfp_ip_r1_1.fastq.gz"

#DRIP-seq
"DRIP\Drip_Meta_2021.xlsx"
"DRIP\at_drip_ndx1-4_r2.bw"
"DRIP\at_drip_ndx1-4_rnaseh.bw"
"DRIP\at_drip_ndx1-4_r1.bw"
```

```
"DRIP\at_drip_col0_rnaseh.bw"
"DRIP\at_drip_ndx1-4.bed"
"DRIP\at_drip_col0_r2.bw"
"DRIP\at_drip_col0_r1.bw"
"DRIP\at_drip_col0.bed"
"DRIP\at_drip_ndx1-4_ip_rnaseh_2.fastq.gz"
"DRIP\at_drip_ndx1-4_ip_rnaseh_1.fastq.gz"
"DRIP\at_drip_ndx1-4_ip_r2_2.fastq.gz"
"DRIP\at_drip_ndx1-4_ip_r2_1.fastq.gz"
"DRIP\at_drip_ndx1-4_ip_r1_2.fastq.gz"
"DRIP\at_drip_ndx1-4_ip_r1_1.fastq.gz"
"DRIP\at_drip_ndx1-4_inp_2.fastq.gz"
"DRIP\at_drip_ndx1-4_inp_1.fastq.gz"
"DRIP\at_drip_col0_ip_rnaseh_2.fastq.gz"
"DRIP\at_drip_col0_ip_rnaseh_1.fastq.gz"
"DRIP\at_drip_col0_ip_r2_2.fastq.gz"
"DRIP\at_drip_col0_ip_r2_1.fastq.gz"
"DRIP\at_drip_col0_ip_r1_2.fastq.gz"
"DRIP\at_drip_col0_ip_r1_1.fastq.gz"
"DRIP\at_drip_col0_inp_2.fastq.gz"
"DRIP\at_drip_col0_inp_1.fastq.gz"

#BS-seq
"GEO_GSE201841_update_with new_SubSeries_0708\BS-seq\BS-seq_Meta_2022.xlsx"
"GEO_GSE201841_update_with new_SubSeries_0708\BS-seq\ndx1-4_vs_col0_DMR.tsv"
"GEO_GSE201841_update_with new_SubSeries_0708\BS-seq\ndx1-4_vs_col0.CG.DMR.bed"
"GEO_GSE201841_update_with new_SubSeries_0708\BS-seq\ndx1-4_vs_col0.CHH.DMR.bed"
"GEO_GSE201841_update_with new_SubSeries_0708\BS-seq\ndx1-4_vs_col0.CHG.DMR.bed"
"GEO_GSE201841_update_with new_SubSeries_0708\BS-seq\at_col0_r1.CHH.bw"
"GEO_GSE201841_update_with new_SubSeries_0708\BS-seq\at_col0_r1.CG.bw"
"GEO_GSE201841_update_with new_SubSeries_0708\BS-seq\at_col0_r1.CHG.bw"
"GEO_GSE201841_update_with new_SubSeries_0708\BS-seq\at_col0_r2.CG.bw"
"GEO_GSE201841_update_with new_SubSeries_0708\BS-seq\at_col0_r2.CHG.bw"
"GEO_GSE201841_update_with new_SubSeries_0708\BS-seq\at_col0_r2.CHH.bw"
"GEO_GSE201841_update_with new_SubSeries_0708\BS-seq\at_ndx1-4_r1.CHH.bw"
"GEO_GSE201841_update_with new_SubSeries_0708\BS-seq\at_ndx1-4_r1.CG.bw"
"GEO_GSE201841_update_with new_SubSeries_0708\BS-seq\at_ndx1-4_r1.CHG.bw"
"GEO_GSE201841_update_with new_SubSeries_0708\BS-seq\at_ndx1-4_r2.CHH.bw"
"GEO_GSE201841_update_with new_SubSeries_0708\BS-seq\at_ndx1-4_r2.CG.bw"
"GEO_GSE201841_update_with new_SubSeries_0708\BS-seq\at_ndx1-4_r2.CHG.bw"
"GEO_GSE201841_update_with new_SubSeries_0708\BS-seq\at_bs_col0_r1_2.fastq.gz"
"GEO_GSE201841_update_with new_SubSeries_0708\BS-seq\at_bs_col0_r1_1.fastq.gz"
"GEO_GSE201841_update_with new_SubSeries_0708\BS-seq\at_bs_col0_r2_1.fastq.gz"
"GEO_GSE201841_update_with new_SubSeries_0708\BS-seq\at_bs_col0_r2_2.fastq.gz"
"GEO_GSE201841_update_with new_SubSeries_0708\BS-seq\at_bs_ndx1-4_r1_2.fastq.gz"
"GEO_GSE201841_update_with new_SubSeries_0708\BS-seq\at_bs_ndx1-4_r1_1.fastq.gz"
"GEO_GSE201841_update_with new_SubSeries_0708\BS-seq\at_bs_ndx1-4_r2_2.fastq.gz"
"GEO_GSE201841_update_with new_SubSeries_0708\BS-seq\at_bs_ndx1-4_r2_1.fastq.gz"

#mRNA-seq
"GEO_GSE201841_update_with new_SubSeries_0708\mRNA-seq\mRNA-seq_Meta_2022.xlsx"
"GEO_GSE201841_update_with new_SubSeries_0708\mRNA-seq\ndx1-4_vs_col0_diff_genes.tsv"
"GEO_GSE201841_update_with new_SubSeries_0708\mRNA-seq\ndx1-4_vs_col0_diff_tes.tsv"
"GEO_GSE201841_update_with new_SubSeries_0708\mRNA-seq\at_rna_col0_r1.bw"
"GEO_GSE201841_update_with new_SubSeries_0708\mRNA-seq\at_rna_col0_r2.bw"
"GEO_GSE201841_update_with new_SubSeries_0708\mRNA-seq\at_rna_ndx1-4_r2.bw"
"GEO_GSE201841_update_with new_SubSeries_0708\mRNA-seq\at_rna_ndx1-4_r1.bw"
"GEO_GSE201841_update_with new_SubSeries_0708\mRNA-seq\at_rna_col0_r2.fastq.gz"
"GEO_GSE201841_update_with new_SubSeries_0708\mRNA-seq\at_rna_col0_r1.fastq.gz"
"GEO_GSE201841_update_with new_SubSeries_0708\mRNA-seq\at_rna_ndx1-4_r2.fastq.gz"
"GEO_GSE201841_update_with new_SubSeries_0708\mRNA-seq\at_rna_ndx1-4_r1.fastq.gz"

#sRNA-seq
"sRNA-seq\sRNA-seq_Meta_2021.xlsx"
"sRNA-seq\srna_col0_r2.fastq.gz"
"sRNA-seq\srna_ndx1-4_r1.fastq.gz"
"sRNA-seq\srna_col0_r1.fastq.gz"
"sRNA-seq\srna_col0_r3.fastq.gz"
"sRNA-seq\srna_ndx1-4_r3.fastq.gz"
"sRNA-seq\srna_ndx1-4_r2.fastq.gz"
"sRNA-seq\ndx1-4_vs_col0_diff_miRNA.tsv"
"sRNA-seq\ndx1-4_vs_col0_diff_sRNA.tsv"

#HiC
"HiC\HiC_Meta_2021.xlsx"
"HiC\at_hic_ndx1-4_r2.hic"
```

```
"HiC\ndx1-4_vs_col0_diff_hic_25kbp.tsv"
"HiC\at_hic_col0.hic"
"HiC\at_hic_ndx1-4_r1.hic"
"HiC\at_hic_ndx1-4_r2_2.fastq.gz"
"HiC\at_hic_ndx1-4_r2_1.fastq.gz"
"HiC\at_hic_ndx1-4_r1_2.fastq.gz"
"HiC\at_hic_ndx1-4_r1_1.fastq.gz"
"HiC\at_hic_col0_2.fastq.gz"
"HiC\at_hic_col0_1.fastq.gz"
```

Genome browser session
(e.g. UCSC)

http://geneart.med.unideb.hu/pub/2021-ndx

Methodology

Replicates

At least two independent biological replicates were used in our study. The peak calling algorithms considered inter-sample variability, however, the replicates were highly correlated with each other (presented in the paper).

Sequencing depth

NGS statistics can be found online in Supplementary Data 1 accompanying the paper.

at_drip_col0_ip_r1	DRIP-seq	I P	Col-0	10-day seedling	paired-end, 150bp	75377482
at_drip_col0_ip_r2	DRIP-seq	IP	Col-0	10-day seedling	paired-end, 150bp	23471522
at_drip_col0_inp	DRIP-seq	input	Col-0	10-day seedling	paired-end, 150bp	39716783
at_drip_col0_ip_rnaseh	DRIP-seq	IP	Col-0	10-day seedling	paired-end, 150bp	17488941
at_drip_ndx1-4_ip_r1	DRIP-seq	IP	ndx1-4	10-day seedling	paired-end, 150bp	77336752
at_drip_ndx1-4_ip_r2	DRIP-seq	IP	ndx1-4	10-day seedling	paired-end, 150bp	27083038
at_drip_ndx1-4_inp	DRIP-seq	input	ndx1-4	10-day seedling	paired-end, 150bp	43914623
at_drip_ndx1-4_ip_rnaseh	DRIP-seq	IP	ndx1-4	10-day seedling	paired-end, 150bp	31413379
at_chip_ndx-flag_ip_r1	ChIP-seq	IP rep1	flag-NDX	10-day seedling	paired-end, 150bp	19632103
at_chip_ndx-flag_ip_r2	ChIP-seq	IP rep2	flag-NDX	10-day seedling	paired-end, 150bp	22053279
at_chip_ndx-flag_inp	ChIP-seq	input	flag-NDX	10-day seedling	paired-end, 150bp	28580252
at_chip_gfp-ndx_ip_r1	ChIP-seq	IP rep1	NDX-GFP	10-day seedling	paired-end, 150bp	7355039
at_chip_gfp-ndx_ip_r2	ChIP-seq	IP rep2	NDX-GFP	10-day seedling	paired-end, 150bp	7889660
at_hic_col0	Hi-C	Hi-C	Col-0	10-day seedling	paired-end, 150bp	201017543
at_hic_ndx1-4_r1	Hi-C	Hi-C	ndx1-4	10-day seedling	paired-end, 150bp	184544537
at_hic_ndx1-4_r2	Hi-C	Hi-C	ndx1-4	10-day seedling	paired-end, 150bp	176249371
at_srna_col0_r1	sRNA-seq	rep1	Col-0	10-day seedling	single-end, 50bp	13324436
at_srna_col0_r2	sRNA-seq	rep2	Col-0	10-day seedling	single-end, 50bp	12733472
at_srna_col0_r3	sRNA-seq	rep3	Col-0	10-day seedling	single-end, 50bp	12196679
at_srna_ndx1-4_r1	sRNA-seq	rep1	ndx1-4	10-day seedling	single-end, 50bp	13974239
at_srna_ndx1-4_r2	sRNA-seq	rep2	ndx1-4	10-day seedling	single-end, 50bp	12329350
at_srna_ndx1-4_r3	sRNA-seq	rep3	ndx1-4	10-day seedling	single-end, 50bp	11816543
at_mrna_col0_r1	mRNA-seq	rep1	Col-0	10-day seedling	single-end, 50bp	19976904
at_mrna_col0_r2	mRNA-seq	rep2	Col-0	10-day seedling	single-end, 50bp	22092568
at_mrna_ndx1-4_r1	mRNA-seq	rep1	ndx1-4	10-day seedling	single-end, 50bp	20787652
at_mrna_ndx1-4_r2	mRNA-seq	rep2	ndx1-4	10-day seedling	single-end, 50bp	22094615
at_bs_col0_r1	BS-seq	rep1	Col-0	10-day seedling	paired-end, 140bp	15334814
at_bs_col0_r2	BS-seq	rep2	Col-0	10-day seedling	paired-end, 140bp	15307128
at_bs_ndx1-4_r1	BS-seq	rep1	ndx1-4	10-day seedling	paired-end, 140bp	16406303
at_bs_ndx1-4_r2	BS-seq	rep2	ndx1-4	10-day seedling	paired-end, 140bp	16824064

Antibodies

S9.6 monoclonal antibody, prepared in house from the S96 hybridoma cell line, was applied for DRIP. Anti-flag antibody - #2044 New England Biolabs; anti-GFP antibody - #ab290 Abcam, were used for ChIP.

Peak calling parameters

Sequenced reads were aligned to the A. thaliana reference genome (TAIR10; NCBI; Ecotype: Columbia-0) using the bowtie2 algorithm. Samtools was used for making .bam files and indexing. Low mapping quality and PCR-duplicated reads were omitted from downstream analysis (http://broadinstitute.github.io/picard/). Deeptools2 bamCoverage was used to create RPKM (Reads Per Kilobase per Million mapped reads) normalized coverage files (.bedgraph and .bigwig). Read densities were calculated for 20 bp bins (--binSize 20 --operation ratio --smoothLength 60 –normalizeUsing RPKM) and IP / input bigwig ratios were made by bigwigCompare. Heatmaps were generated for 10 bp bins with computeMatrix and were plotted by plotHeatmap functions of deepTools2. For metagene profiles, mean normalized read coverage were calculated in 20 bp windows with computeMatrix and were plotted using plotProfile (deepTools2). MACS2 was used with default settings to identify ChIP peaks in input normalized flag-NDX, NDX-GFP, and DRIP peaks in input normalized and RNaseH-corrected Col-0 and ndx1-4 samples, respectively.

```
macs2 bdgpeakcall -i ${bgPath}at_drip_col0_IPperINP.bg -c 5 -l 200 --no-trackline --outdir ${tempPeakPath} -o
at_drip_col0_IPperINP.bed
macs2 bdgpeakcall -i ${bgPath}at_drip_ndx14_IPperINP.bg -c 5 -l 200 --no-trackline --outdir ${tempPeakPath} -o
at_drip_ndx14_IPperINP.bed
for i in at_chip_flag-ndx_rpl1 at_chip_flag-ndx_rpl2 at_chip_flag-ndx_concat at_chip_ndx-gfp_rpl1 at_chip_ndx-gfp_rpl2
at_chip_ndx-gfp_concat
do
  macs2 bdgpeakcall -i ${bgPath}${i}.bedgraph -c 8 -l 100 --no-trackline --outdir ${peak3Path} -o ${i}.bed
  macs2 bdgpeakcall -i ${bgPath}${i}.rm_d.bedgraph -c 8 -l 100 --no-trackline --outdir ${peak3Path} -o ${i}.rm_d.bed
done
```

Data quality

There are 2243 flag-NDX, 543 NDX-GFP, and 14124 DRIP-seq peaks at <=5% FDR and 100% of the peaks included in the published list

Data quality	showed > 5-fold enrichment compared to local background.
Software	bowtie2 2.3.5.1 for ChIP-seq, DRIP-seq alignments<br>samtools 1.7 for making .bam files and indexing in ChIP-seq, DRIP-seq experiments<br>picard 2.13.2 for filtering low mapping quality and PCR-duplicated reads in NGS experiments<br>deeptools 3.3.1 to create RPKM (Reads Per Kilobase per Million mapped reads) normalized coverage files (.bedgraph and .bigwig)<br>MACS v2.2.7.1 to identify ChIP and DRIP peaks<br>bedtools v2.27.1 for genomic annotation of DRIP and ChIP peaks<br>R-project v4.1 for statistical calculations and figure generations<br>Cutadapt 3.4 to trim Illumina adapters in NGS experiments<br>JBrowse 1.6.11 for genome browser representation of ChIP-seq and DRIP-seq data
